# 

**Published:** 1786

**Authors:** 


### LONDON MEDICAL JOURNAL.

VOLUME THE SEVENTH.

FOR THE YEAR 1786.

Surgeon General's Office LIBRARY. 63446 Washington, D.C.

LONDON:

PRINTED for the EDITOR,

And ſold by JOSEPH JOHNSON, LONDON; CHARLES ELLIOT, EDINBURGH; And P. BYRNE, DUBLIN.

M,DCC,LXXXVI.

### ADVERTISEMENT.

IN proportion as the number of our correſpondents has increaſed, that part of the work which relates to new publications has been neceſſarily contracted within narrower limits. Some of our readers, who have obſerved this increaſe of our correſpondence, and the many uſeful and intereſting facts it has enabled us to preſent to the public, have expreſſed a wiſh to ſee the journal confined ſolely to original papers: but as the ſupply of materials from this ſource muſt be liable to great uncertainty, the propoſed alteration, we fear, would hardly fail to deprive the work of the advantage it ſeems at preſent to derive from the regularity of its appearance at ſtated periods. We ſhall therefore continue to collect a part of our materials from the tranſactions of learned ſocieties, and other printed works; but without profeſſing to give a general review of medical books. Of theſe, however, we ſhall, as uſual, inſert a catalogue.

Communications for this work may be addreſſed to Dr. Simmons, Air Street, Piccadilly, London.

### CATALOGUE OF BOOKS.

1. JOURNAL de Medecine de Londres, pour l'Année 1785, traduit * de l'Anglois de M. *Samuel Foart Simmons*, Medecin de Londres; par M. *Masuyer*, D. M. de l'Université de

* In a well-written dedication of this translation of the London Medical Journal to M. Amelot de Chaillou, Intendant of Burgundy, the learned translator is pleased to offer the de Montpellier, Agregé au College des Medecins de Dijon, & Aſſocié a l'Academie Royale des Sciences, Arts, & Belles Lettres de cette Ville, &c. 8vo. Dijon, 1785.

2. Elements of Mineralogy, by *Richard Kirwan*, Eſq. F. R. S. 8vo. *Elmſley*, London, 1784.

3. Experiments and Obſervations on a new ſpecies of Bark, ſhewing its great Efficacy in very ſmall Doſes*; alſo a comparative View of the Powers of the Red and Quilled Bark; being an Attempt towards a general Analyſis, and compendious Hiſtory of the valuable Genus of Cinchona or the Peruvian Bark. By the following, among other obſervations, in ſupport of the utility of his undertaking. " The practice of the Engliſh " phyſicians," ſays he, " is ſo different from ours, and their " doctrines ſo much unlike thoſe we have been accuſ- " tomed to adopt, in an infinite number of circumſtances, " that medical ſcience muſt neceſſarily derive great advan- " tages from diſcuſſions, which, it is hoped, will tend gra- " dually to remove this contrariety of opinions, and to bring " two of the moſt enlightened nations of Europe to a more " uniform manner of thinking on theſe ſubjects." — M. Maſuyer has thought it right to commence his publication with the volume for the year 1785, but he is preparing a tranſlation of all the preceding volumes.

* See Vol. VI. p. 175.

Richard

*Richard Kentiſh*, M. D. Member of the Royal Medical Society at Edinburgh, Correſpondent Member of the Society of Scottiſh Antiquaries, &c. 8vo. *Johnſon*, London, 1785.

4. An Eſſay on the Theory of the Production of Animal Heat, and on its Application in the Treatment of cutaneous Eruptions, Inflammations, and ſome other Diſeaſes. By *Edward Rigby*, Member of the Corporation of Surgeons in London. 8vo. *Johnſon*, London, 1785.

5. A New Experimental Inquiry into the Nature and Qualities of the Cheltenham Water; with a conciſe Account of the Diſeaſes wherein it is chiefly indicated; and the Diet and Regimen neceſſary to its ſucceſsful Uſe. By *A. Fothergill*, M. D. F. R. S. Member of the Royal College of Phyſicians in London, of the Medical Societies of London and Edinburgh; and Phyſician in Bath. 8vo. *Cruttwell*, Bath, 1785.

6. The Structure and Phyſiology of Fiſhes explained, and compared with thoſe of Man and other Animals. Illuſtrated with Figures. By *Alexander Monro*, M.D. Fellow of the Royal College of Phyſicians and of the Royal Society, and Profeſſor of Phyſic, Anatomy, and Surgery, gery, gery, in the University of Edinburgh. Folio. *Elliot*, Edinburgh, 1785.

7. Filices Britannicæ; an History of the British proper Ferns; with plain and accurate Descriptions, and new Figures of all the Species and Varieties, taken from an immediate and careful Inspection of the Plants in their natural State, and engraved on thirty-one Copper-plates; with the particular Places noted where each Species was lately gathered, and is at this time growing in the North of England, or on the Mountains of Wales. By *James Bolton*, of Halifax. 4to. Leeds, 1785.

8. Medical Sketches. Part the First. By *Richard Pew*, Member of the Royal Society at Edinburgh. 8vo. *Bew*, London, 1785.

9. Nosologia Methodica Oculorum, or a Treatise on the Diseases of the Eyes; selected and translated from the Latin of *Francis Boissier de Sauvages*; wherein the whole are methodically arranged; to which are also added the Description and Methods of Cure, recited by those Authors who have written professedly on the various Subjects herein enumerated. By *George Wallis*, M. D. 8vo. *Robinson*, London, 1785.

10. A Differtation on Milk. In which an attempt is made to afcertain its natural ufe; to inveftigate experimentally its general nature and properties; and to explain its effects in the cure of various difeafes; likewife to point out the varieties in the food of the animal from which it is taken; and the circumftances in the mode of life, and conduct of thofe women who afford it, which more efpecially tend to change its appearance, and to impair its falutary qualities; and particularly to enforce the cautions and reftrictions which are neceffary to be obferved by thofe, whofe duty or bufinefs it is to fuckle an infant race. By *Samuel Ferris*, M. D. Extraordinary Member, and late Prefident of the Royal Medical Society at Edinburgh. 8vo. *Cadell*, London, 1785.

11. Directions for impregnating the Buxton Water, with its own and other Gafes; and for compofing artificial Buxton Water. By *George Pearfon*, M. D. Member of the Royal College of Phyficians, London. 8vo. London, 1785.

12. An Effay on the Virulent Gonorrhœa; in which the different opinions refpecting the treatment of the difeafe are carefully examined, and a method of cure deduced from them, as founded on the principles of Anatomy and Phyfiology.

ſiology. By *J. Clubbe*, Surgeon. 8vo. *Murray*, London, 1786.

13. Aretæus; conſiſting of eight Books, on the Cauſes, Symptoms, and Cure of acute and chronic Diſeaſes, tranſlated from the original Greek. By *John Moffat*, M. D. 8vo. *Richardſon*, London, 1786.

14. A Treatiſe upon Gout: in which the primitive cauſe of that Diſeaſe, and likewiſe of Gravel, is clearly aſcertained; and an eaſy Method recommended, by which both may be with certainty prevented, or radically cured. 8vo. *Cadell*, London, 1786.

15. An Account of an entire new Method of curing Burns and Scalds. 8vo. *Fielding*, London, 1786.

16. Arbuſtrum Americanum; the American Grove, or an alphabetical Catalogue of Foreſt Trees and Shrubs, natives of the American United States, arranged according to the Linnæan Syſtem; containing the particular diſtinguiſhing character of each genus, with plain, ſimple, and familiar deſcriptions of the manner of growth, appearance, &c. of their ſeveral ſpecies and varieties: alſo ſome hints of their uſes in Medicine, Dyes, and Domeſtic Oeconomy. Compiled from actual knowledge and obſervation, and the aſſiſtance of botanical authors. By *Humphry Marſhall.* 8vo. Philadelphia, 1785.

17. Explanation and Index of two Mineralogical Tables *. By *Tiberius Cavallo*, F.R.S. 8vo. *Dilly*, London, 1786.

18. Scriptorum Latinorum de Aneuryſmatibus Collectio †. Edidit atque præfatus eſt *Thomas Lauth*, M. D. et Prof. P. cum 15 iconibus. 4to. Argentorati, 1785.

19. *Edvardi Sandifort* Medicinæ, Anatomes, et Chirurgiæ, in Academia Batava, quæ Leidæ eſt, Profeſſoris, Deſcriptio Oſſium Hominis. Accedit Oratio de officio Medici perquam difficili,

* The firſt of the tables which accompany this Pamphlet, contains all the minerals hitherto diſcovered, arranged in claſſes, orders, genera, ſpecies, and varieties; together with the proportion of the ingredients, or primitive bodies, of which they are compoſed. The ſecond table contains the principal characteriſtic properties of minerals, and ſeems to be fully ſufficient for the purpoſe of indicating the names or principal properties of theſe ſubſtances.

† The works which the learned Editor has collected in this volume are—J. M. Lanciſii de Aneuryſmatibus opus poſthumum—Car. Guattani opus de Aneuryſmatibus—Ant. Matani de Aneuryſmaticis Præcordiorum Morbis Animadverſiones—

cili, a multis pessime neglecto. 4to. Lugduni Batavorum, 1785.

20. Elemens de Medecine Pratique, de M. *Cullen*, M. D. traduits de l'Anglois sur la 4ieme et derniere edition, avec des notes, par M. *Bosquilion*, Ecuyer, Docteur Regent de la Faculté de Medecine, &c. Tome Ier, 8vo. Paris, 1785.

21. Institutions de Medecine Pratique, traduits sur la quatrieme et derniere Edition de l'ouvrage Anglois de M. *Cullen*, Professeur de Medecine Pratique dans l'Université d'Edinbourg, des Societés Royales de Londres, d'Edinbourg, &c. Premier Medecin du Roi pour l'Ecosse. Par M. *Pinel*, D. M. Tomes II. 8vo. Paris, 1785.

22. Observations sur la Cure de la Gonorrhée, traduites* de l'Anglois de M. *Samuel Foart*

siones— Jacobi Verbrugge Diss. Anat. Chirurgica de Aneurysmate — Joh. Jac. Weltini Dissert. Inauguralis Medica de Aneurysmate vero Pectoris externo Hemiplegiæ sobole — Adolph. Murray in Aneurysmata Femoris Observationes — Christ. Jacobi Trew Aneurysmatis spurii post Venæ Basilicæ sectionem orti Historia et Curatio — Conradi Asman Dissert. Medica Inaug. de Aneurysmate.

* A former translation of this little work into French was announced in Vol. iv. p. 321. To the present translation M. Masuyer has added some valuable notes.

*Simmons*,

*Simmons*, Docteur en Medecine, de la Societé Royale de Londres, Membre du College Royal des Medecins de la même Ville; et de la Societé Royale de Medecine de Paris. Par *Gabriel Masuyer*, D. M. 8vo. Montpellier, 1784.

23. Observaciones sobre la Curacion de la Gonorrea, de D. *Samuel Foart Simmons*, Doctor en Medicina, Miembro del Colegio Real de los Medicos, y de la Sociedad Real de Londres; Asociado Extrangero de la Sociedad Real de Medicina de Paris, &c. Traducidas por D. *Francisco Xavier de Cascaron*, Cirujano del Real Sitio de Buenretiro, y de la Real Familia. 12mo. Madrid, 1784.

24. Heelkungige Mengelstoffen: *i. e.* Chirurgical Miscellanies. By *G. J. Van Wy*, surgeon to the hospital at Amsterdam. 8vo. Amsterdam, 1784.

25. Anatomische und Chirurgische Anmerkungen, &c. *i. e.* Anatomical and Chirurgical Observations. By *Frederick Lebegolt Pitschel*, M. D. professor of Anatomy, and physician to the garrison at Dresden. 8vo. Dresden, 1784.

26. Introductio in Oryctographiam et Zoologiam Arragoniæ; accedit Enumeratio Stirpium in eadem regione noviter detectarum. 8vo. Saragossa, 1784.

27. Curso

27. Curſo Elemental de Botanica, Teorico y Practico, diſpueſto para la Enſenanza del Real Jardin Botanico de Madrid de orden del Rey Nueſtro Senor, por el Dr. D. *Caſimiro Gomez Ortega*, y D. *Antonio Palau y Verdera*, Catedraticos primero y ſegundo del miſmo Jardin. 8vo. Madrid, 1785.

28. *Caroli Petri Thunberg*, Med. Doct. Prof Reg. et Extraord. Academ. Cæſ. N. C. Reg. Scient. Holmens. Societ. Litter. Upſal. Patriot. Holmens. Berolin. N. Scrut. Lundin. Harlemens. Amſtedam. Nidroſiens. Membri, Flora Japonica, ſiſtens Plantas Inſularum Japonicarum ſecundum Syſtema ſexuale emendatum redactas ad xx Claſſes, Ordines, Genera et Species cum differentiis ſpecificis, ſynonymis paucis, deſcriptionibus concinnis, et xxxix iconibus adjectis. 8vo. Lipſiæ, 1784.

29. Theoria Generationis et fructificationis plantarum cryptogamicarum mere propriis; obſervationibus et experimentis ſuperſtructa: Diſſertatio quæ præmio ab Academia Imperiali Petropolitana, pro anno, 1783, propoſito ornata eſt. Auctore *Joanne Hedwig*. M. D. Societatis Phyſiophilorum Berolinenſis, et Œconomiæ Lipſienſis ſocio. 4to. Petropoli, 1784. cum tabulis æneis.

30. Diſ-

30. Differtatio Medico-Chirurgica de Fonticulorum ufu in Sanandis Morbis. Auctore *Joh. P. Ham.* 4to. Argentor. 1784.

31. Differtatio Inauguralis-Medica de Hydrope Cyftico. Auctore *Theop. Guft. Eifenlohr.* 4to. Argentor. 1784.

32. Memoires de Chymie, de M. *C. W. Scheele*, tirés des Memoires de l'Academie Royale des Sciences de Stockholm, traduits du Suedois et de l'Allemand. 12mo. Dijon, 1785.

This volume, for which the public are indebted to Madame Picardet, of Dijon, contains a tranflation of twenty-feven chemical effays, by Mr. Scheele. The notes, which are extremely valuable, are by M. de Morveau.

33. Pharmacopeia Wirtenbergica, in duas partes divifa; quarum prior Materiam Medicam hiftorico—phyfico — medice defcriptam, pofterior compofita et præparata, modum præparandi et encheirefes exhibet, curâ Collegii Archiatralis Wirtenbergici. Editio nova, revifa, aucta et emendata. 8vo. Laufanne, 1785.

34. Differtatio Inauguralis de ufu aquæ frigidæ in hæmorrhagiis Uteri. Auctore *Franc. Joach. Puttmann*, Hildefienfi. 4to. Argentorati, 1785.

